# The Effects of Active Video Games on Health-Related Physical Fitness and Motor Competence in Children and Adolescents with Healthy Weight: A Systematic Review and Meta-Analysis

**DOI:** 10.3390/ijerph18136965

**Published:** 2021-06-29

**Authors:** Cristina Comeras-Chueca, Jorge Marin-Puyalto, Angel Matute-Llorente, German Vicente-Rodriguez, Jose A. Casajus, Alex Gonzalez-Aguero

**Affiliations:** 1 Department of Physiatry and Nursing, Faculty of Health Science, Universidad de Zaragoza, 50009 Zaragoza, Spain; ccomeras@unizar.es (C.C.-C.); joseant@unizar.es (J.A.C.); 2 GENUD (Growth, Exercise, Nutrition and Development) Research Group, 50009 Zaragoza, Spain; jmarinp@unizar.es (J.M.-P.); amatute@unizar.es (A.M.-L.); gervicen@unizar.es (G.V.-R.); 3 EXERNET, Red de Investigación en Ejercicio Físico y Salud Para Poblaciones Especiales, 50009 Zaragoza, Spain; 4 Department of Physiatry and Nursing, Faculty of Health and Sport Science (FCSD), Universidad de Zaragoza, 22001 Huesca, Spain; 5 Instituto Agroalimentario de Aragón—IA2—(CITA-Universidad de Zaragoza), 50009 Zaragoza, Spain; 6 Centro de Investigación Biomédica en Red de Fisiopatología de la Obesidad y Nutrición (CIBERObn), 50009 Zaragoza, Spain

**Keywords:** exergames, fitness, motor competence, youth, Pediatric Inactivity Triad

## Abstract

(1) Background: Poor levels of physical fitness and motor skills are problems for today’s children. Active video games (AVG) could be an attractive strategy to help address them. The aim was to investigate the effects of AVG on health-related physical fitness and motor competence in children and adolescents with healthy weight. (2) Methods: Randomized and non-randomized controlled trials investigating the effects of AVG programs on health-related physical fitness and motor competence were included. Two different quality assessment tools were used to measure the risk of bias. Twenty articles met the inclusion criteria and the variables of interest were body mass index (BMI), body fat, cardiorespiratory fitness (CRF), muscular fitness and motor competence. (3) Results: AVG interventions seem to have benefits in BMI when lasting longer than 18 weeks (SMD, −0.590; 95% IC, −1.071, −0.108) and in CRF (SMD, 0.438; 95% IC, 0.022, 0.855). AVG seems to be a promising tool to improve muscular fitness and motor competence but the effects are still unclear due to the lack of evidence. (4) Conclusions: AVG seem to be an effective tool for improving some components of health-related physical fitness and motor competence in healthy-weight children and adolescents, but the effect on some fitness components needs further research. Therefore, AVG may be included as a strategy to improve health.

## 1. Introduction

Childhood and adolescence are decisive periods because numerous physical, physiological and psychological changes take place [1]. In fact, lifestyle, understood as the habits and behaviors that, whether healthy or not, are acquired during these stages and have a clear influence on adult health [1,2]. Physical activity (PA) is generally considered a healthy lifestyle directly related to health but also to physical fitness [3,4]; being also true that although fitness has an important genetic component, it is also associated with active and sedentary behaviors [4,5].

Health-related physical fitness is currently considered one of the most important health markers [1,3]. The level of physical fitness is a predictor of morbidity and mortality for cardiovascular and metabolic disease and for all causes of death in life [1,4,5,6]. Improving health-related physical fitness, mainly cardiovascular and muscular fitness and body composition, is a means to improve health [2].

Children with a low cardiorespiratory fitness (CRF) level are more likely to exceed the healthy weight body mass index (BMI) status [2]. This is important because childhood obesity is one of the most important public health problems of the 21st century [7] and prevalence of childhood overweight and obesity has acquired the status of an epidemic; for instance, in 2016, the prevalence of overweight was over 30% and the prevalence of obesity was over 10% in European children and adolescents [8]. It therefore seems that a good early obesity prevention strategy could be to increase the levels of PA [9] and physical fitness [10] of young people, which are anyway interrelated and will also have an impact on each other [4].

PA is one of the most important factors in CRF [4] and it has a great influence on body composition [9]. PA has been widely and favorably associated with body fat, cardiometabolic biomarkers, physical fitness, bone health, motor competence and, in short, the quality of life; and insufficient PA is one of the main risk factors for non-communicable diseases and for premature death [11]. The recommendation of WHO indicates that an average of 60 min of moderate-to-vigorous PA daily provides benefits for health in young people, although PA beyond 60 min of moderate-to-vigorous PA daily provides additional benefits [12]. However, a large proportion of the children and adolescents do not meet the public health recommendations of PA, as showed in 2016, in a study of 1.6 million students aged 11–17 in which 81.0% of them did not meet this recommendation [13].

Physical inactivity in youth is a serious problem in today’s society and is included in the Pediatric Inactivity Triad (PIT) by Faigenbaum et al. [14], which comprised three inter-related components: exercise deficit disorder, pediatric dynapenia, and physical illiteracy. The exercise deficit disorder is the term used to describe those children and adolescents that do not meet the current public health recommendations of PA and this term pretend “to highlight the gravity of this clinical condition” [14]. This exercise deficit disorder often leads to an excessive increase in body fat and a low fitness level [11].

The PIT notes the importance of muscular endurance and strength. Scientific evidence shows a trend of decline in muscle fitness compared to previous years [15,16,17]. Therefore, global trends indicate that children have a poorer level of muscular fitness than previous generations, which is related to a lower level of motor skills, PA and organized-sports participation [18,19]. A systematic review about trends in muscular fitness between 1972 and 2015 showed small increases in the relative muscular strength and speed while proxies of muscle power declined [20]. Muscular fitness is important in the development of children because “strength is the staple that holds other fitness components together” [18], together with its relationship with motor competence [14,20].

Further, the PIT highlights highlight the relevance of motor competence [14], understand as motor skill proficiency. The primary school years are considered the key stage to develop motor competence and to adopt an active or inactive lifestyle [21]. Motor competence positively correlated with PA and health-related physical fitness [22,23,24], which reinforces the importance of including the improvement of motor competence in the main objectives of physical exercise interventions for children and adolescents [25].

The decreased levels of PA and health-related physical fitness in children and adolescents are associated with adverse consequences on their health such as obesity, type II diabetes, cardiovascular and metabolic diseases, etc. [6] It is well known that exercise is an effective tool to obtain all the holistic benefits of exercise, such as improvements in BMI status or adiposity, in cardiorespiratory and muscular fitness or in bone health [26]. However, the main challenge is to ensure adherence to exercise [27], especially in the young population with particular possibilities of low practice.

Active-video games (AVG) have been proposed as a good alternative aiming to motivate those (young or not) who find sport less interesting, partly due to their body composition, poor performance or low motor skills. Scientific literature has shown the potential of AVG to increase the energy expenditure and to replace sedentary behaviors by promoting light-to-moderate PA [28,29,30,31,32,33,34]. Although some studies have reported the benefits of AVG on body composition [35], CRF [36], and motor competence [37,38], the effects of AVG on health-related physical fitness need further investigation because of the importance it can have for their present and future health.

It is therefore necessary to ascertain the potential of AVG for improving health in children and adolescents with healthy weight and for promoting a healthier lifestyle. As well, this update of the evidence is necessary as AVGs have been updated, offering more and more possibilities and increasing the physical demands in exercise sessions through AVG [34]. Consequently, this systematic review aims to identify and critically appraise current research results from the data obtained and to pool the results of published studies in a meta-analysis. This will allow us to provide a clear answer on the effects of AVG on health-related physical fitness and motor competence in children and adolescents with healthy weight. To define children with a healthy weight, the BMI zscore values were from >−2 to ≤1 or the BMI percentile from >3rd percentile up to 85th percentile [39].

## 2. Materials and Methods

This review has been performed following the criteria and methodology established by Cochrane Handbook for Systematic Reviews of Interventions (Version 5.1.0) [40]. The writing of this review was performed according to the Preferred Reporting Items for Systematic reviews and Meta-Analyses (PRISMA) 2020 statement [41]. PRISMA 2020 item Checklist has been included as a supplementary file (Table S1). A protocol was registered in the International Prospective Register of Systematic Reviews, PROSPERO (CRD42020222831).

### 2.1. Data Sources and Search Strategy

Journal articles were identified by searching electronic databases, scanning reference lists of articles and examining tables from earlier systematic reviews. The search strategy was applied to PubMed, Medline, Web of Science and SPORTDiscus up to and including March 2021.

The search strategy used to identify the articles in PubMed and Medline was as follows: exergam* OR “active video gam*” OR “active videogam*” OR “active gam*” OR “interactive video gam*” OR “interactive videogam*” OR “Wii” OR “Xbox” OR “Kinect” OR “PlayStation”, and “Species: Humans” and “Language: English” filters were applied, along with “Journal Article” for Medline. The search strategy applied in SPORTDiscus was as follows: TX = (exergam* OR “active gam*” OR “active video gam*” OR “active videogam*” OR “interactive video gam*” OR “interactive videogam*” OR “Wii” OR “Xbox” OR “Kinect” OR “PlayStation”) and “document type: article” and “language: English” filters were applied. The search strategy used in Web of Science was as follows: TS = (exergam* OR “active gam*” OR “active video gam*” OR “active videogam*” OR “interactive video gam*” OR “interactive videogam*” OR “Wii” OR “Xbox” OR “Kinect” OR “PlayStation”) and “document type: article” and “language: English” filter was applied.

Two reviewers (C.C.C. and A.G.A.) independently evaluated all studies. Titles and abstracts were examined, and relevant articles were obtained and assessed using the inclusion and exclusion criteria described below. Inter-reviewer disagreements were resolved by consensus. A third reviewer resolved disagreements.

### 2.2. Inclusion Criteria

The following inclusion criteria were used following PICOS format [42]: (P) participants: under 18 years old without overweight or obesity; (I) trials studying the effects of AVG programs on health-related physical fitness and motor competence; (C) control group with no intervention or with traditional exercise intervention; (O) types of outcome measures: variables of health-related physical fitness such as CRF, musculoskeletal fitness (muscular strength and muscular endurance) and body composition, and variables related with motor competence; (S) types of study: randomized and non-randomized controlled.

### 2.3. Exclusion Criteria

The following exclusion criteria were applied: (1) studies in languages aside from English or Spanish; (2) unpublished data; (3) studies with animals; (4) studies including participants with disabilities, diseases or disorders; (5) studies without pre- and post-assessments of the variables of interest (6) dissertations or abstracts from society proceedings or congresses.

### 2.4. Search Summary

**Figure 1:**
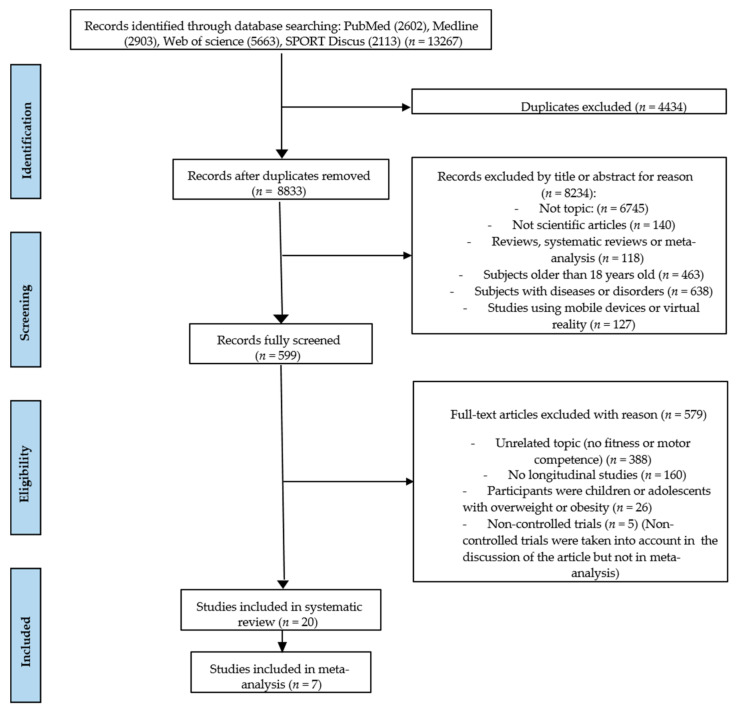
PRISMA flow diagram of articles that were selected.

A total of 13267 relevant articles were identified using the search strategies. Following a review of titles and abstracts, and excluding duplicates, the total number of articles was reduced to 599. Then, 20 articles met the inclusion criteria and were selected to be included in this review. Articles were excluded because of the following reasons: (1) cross-sectional studies (*n* = 160), (2) only psychological, cognition, nutrition, balance variables, PA or energy expenditure were measured (*n* = 388), (3) studies that included children and adolescents with overweight and obesity (*n* = 26), (4) and non-controlled trials (*n* = 5) (Figure 1).

The characteristics of each study included in this systematic review were summarized in different sections following PICOS format [42]: participants (P), intervention (I), comparison between groups or control group (C), outcomes (O), and study design (S).

### 2.5. Risk of Bias

Two risk-of-bias assessment tools were used for assessing risk of bias proposed in the PRISMA 2020 statement, the RoB 2 in randomized controlled trials updated by Sterne et al. [43] and the ROBINS-I (“Risk of Bias in Non-randomized Studies—of Interventions”) in non-randomized controlled trial developed by Sterne et al. [44].

### 2.6. Data Extraction

The following information was extracted from each included trial: name of first author, year of publication, sample size, participant characteristics including age, sex and BMI status, type of study, type of intervention, training characteristics including intervention length and frequency, variables and data sources and outcomes. From this point in the manuscript onwards, when not specifying the activity performed by the control group, this will imply that the control group continued with their normal routine.

### 2.7. Meta-Analyses

Children and adolescents who performed an AVG intervention were compared with controls who participated in no intervention or in another type of PA intervention. Effect sizes were calculated for each outcome (BMI, body fat percentage, CRF, waist circumference, fat-free mass, muscular fitness and motor competence). Different meta-analyses were performed stratifying by type of control group (no intervention or PA intervention without AVGs).

Standardized mean differences (SMD) between participants in AVG interventions and controls and their 95% confidence intervals were calculated using a continuous random-effects model (DerSimonian–Laird method). SMD effects sizes were interpreted as follows: <0.40 = small, 0.40–0.70 = moderate, >0.70 = large effect [40]. The heterogeneity of the studies was tested using the I^2^ statistic [45]. This statistic describes the variance between studies as a proportion of the total variance and was interpreted as follows: I^2^ = 0–25% no heterogeneity, I^2^ = 25–50% moderate heterogeneity, I^2^ = 50–75% high heterogeneity, and I^2^ = 75–100% very high heterogeneity. All analyses were performed using the Open Meta [Analyst] software (Windows 10, Rhode Island, RI, USA).

## 3. Results and Discussion

Table 1 summarizes the details of the methodological quality assessment for randomized controlled trials. The risk of bias for randomized controlled trial was low.

Table 2 summarizes the details of the methodological quality assessment for the one non-randomized controlled trial, showing low risk of bias.

### 3.1. AVG Interventions

There was a great deal of variety across AVG used. Interventions mostly ran during Physical Education (PE) lessons, during playtime or lunchtime, or as an extracurricular activity after school. Most used devices in AVG interventions were gaming consoles such as Xbox 360 with Kinect^®^, Nintendo Wii^®^, Sony PlayStation 2^®^, dance mats, and also other games such as (GoNoodles^®^, “Adventure to Fitness” and “Cosmic Kids Yoga”.

The length of AVG interventions ranged from 6 weeks to 12 months (mean: 16.1 ± 13.2 weeks). Frequency of AVG sessions ran from 2 to 7 days per week (mean: 3.8 ± 1.7 days per week), although some interventions were applied daily. Sessions typically lasted between 10 and 60 min (mean: 44.3 ± 26.0 min) and were delivered by PE teachers and research assistants or, in other cases, they were developed autonomously (Home-based AVG interventions). It is therefore complicated to establish a standard length, intensity, duration of sessions or type of AVG interventions.

The different control groups either were applied another intervention without AVG, like PE or exercise sessions, access to sedentary video games, learning sessions, or were just asked to continue their normal activities of daily life, the latter being the most used option for the control group.

### 3.2. AVG Effects

All the studies concerning the effects of AVG on health-related physical fitness and motor competence in children and adolescents with healthy weight are summarized in Table 3. A total of twenty randomized and non-randomized controlled trials showed effects of AVG on health-related physical fitness and motor competence in children and adolescents with healthy weight [35,36,37,38,46,47,48,49,50,51,52,53,54,55,56,57,58,59,60,61]. Although the non-controlled articles were not taken into account in the results, they were reviewed in the discussion and were summarized in Table S2.

**Figure 2:**
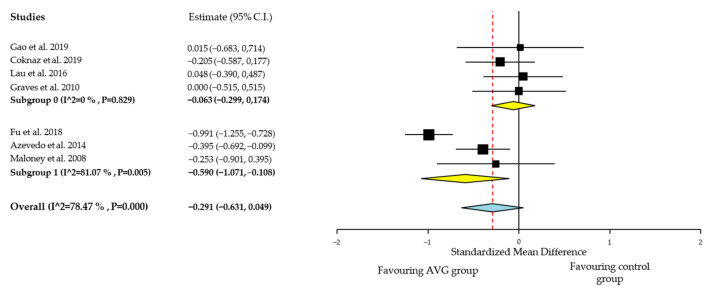
BMI effect sizes for AVG compared with control group. Subgroup analysis by AVG intervention’s length; Subgroup 1: AVG interventions lasting less than 18 weeks; Subgroup 2: AVG interventions lasting 18 weeks or more.

Individual study results and global effects of quantitative effects of CRF are plotted in Figure 2, while a summary of the global result is presented in Table 4. Some articles were excluded from the quantitative analyses given that the effect sizes could not be calculated from the information available in the papers [48,50].

#### 3.2.1. Body Mass Index and Percentage of Body Fat

BMI, fat mass or body fat percentage were evaluated by eight articles [35,38,48,49,51,57,58,59] and fat mass or body fat percentage were measured by dual-energy X-ray absorptiometry (DXA) [35,58]. Four out of eight studies reported that an AVG intervention decreased BMI in children with healthy weight [35,38,57]. Azevedo et al. [35] investigated the effect of an AVG with dance mats and the results showed a positive intervention effect on weight (−1.7 kg, 95% CI: −2.9 to −0.4) and BMI (−0.9 kg/m^2^, 95% CI: −1.3 to −0.4) compared to a control group. A similar AVG was used by Maloney et al. [57], displaying a higher decrease in mean BMI percentile by children who participated in AVG in comparison with a control group (5.6 vs. 0.2 BMI percentile decrease, respectively). Coknaz et al. [49] investigated the effect of a 12-week AVG intervention with Nintendo Wii and positive intervention effects on BMI were observed. Ye et al. [38] examined the effectiveness of a combined AVG and PE program using Xbox Kinect and Nintendo Wii compared to PE only, across a school year with 125 min weekly, showing that AVG had positive effects on BMI (−0.3 kg/m^2^), while PE control group increased it (1.28 kg/m²).

On the other hand, four randomized controlled trials reported no effects [48,58,59] or negative effects [51], three of these were for non-supervised home-use [48,58,59] and another three of these lasted less than 12 weeks [48,51,58]. The first study on the effect of AVG on BMI was performed by Maloney et al. [59]. AVG group were provided with all equipment necessary to play DDR at home but there were no changes in BMI in neither AVG nor control groups. The same practice was followed by Graves et al. [58]. These authors reported no AVG effects on BMI after 12 weeks of non-supervised home-use Nintendo Wii. Gao et al. [48] showed no effects and no differences between AVG and control groups. Lau et al. [51] showed an increase in BMI (*p* < 0.001), while this increase was not shown in control group after a 12-week intervention with Xbox Kinect. Negative effects of the AVG group relative to control group were reported (0.22 kg/m^2^, 95% CI: −0.46 to 0.90).

Only two studies reported effects of AVG on body fat in healthy-weight children. Azevedo et al. [35] showed a positive effect on body fat with a 1-year AVG intervention with dance mats compared to control group (−2.2%, 95% CI: −4.2 to −0.2). However, Graves et al. [58] reported no effects on body fat in AVG or control group after a home-based AVG intervention with Nintendo Wii over 12 weeks.

Non-supervised home-based AVG interventions do not seem to produce enough stimulus to have an effect on the BMI or percentage of body fat. De Brito-Gomes et al. [62] showed that higher energy expenditure was observed in structured interventions.

Two non-controlled studies also reported BMI and body fat changes after AVG intervention [63,64]. Bethea et al. [64] applied a 30-week AVG intervention with DDR and no changes were shown in BMI. These results were similar to those found in the article performed by Owens et al. [63] that evaluated the change in BMI and body fat percentage after a 3-month AVG intervention in which participants were provided with a Nintendo Wii to play at home, with no effects shown. It is worth noting that the intervention in this study was family-based and that they spent 12.6 min per day on average but that there was a significant reduction in Wii time between the first six and last six weeks of the intervention.

Some systematic reviews studied the effects of AVG on BMI or body fat, including studies of overweight or obese children or adolescents., but some limitations can be found, such as the inclusion of non-controlled trials, the inclusion of children and adolescents with diseases or disorders, or the search strategy, or differences in the inclusion and exclusion criteria such as including children and adolescents with overweight and obesity [31,65,66,67,68,69,70,71]. Our results are in line with theirs. The latest review, by Gao et al. [65], included studies in children with obesity or overweight, and the results showed improvements in body composition after AVG interventions. Hernández-Jimenez et al. [66] performed a meta-analysis showing a significant effect in favor of AVG on BMI in children and adolescents, with greater results when the AVG intervention was applied to overweight or obese children. Oliveira et al. [67] performed a systematic review focused on healthy-weight children and adolescents and the results showed that AVG were effective in reducing weight and BMI. Another systematic review [68] showed that most of the randomized controlled trials reported that AVG interventions had a positive effect on BMI, body composition, or body fat. A previous systematic review performed by Gao et al. [69] not focused on obesity, concluded that AVG were a promising tool to promote PA and health, as long as the AVG intervention is not home-based. In the systematic review by Norris et al. [70] which included some articles with obese children, half of the included articles assessing BMI or body composition reported positive effects following AVG intervention while the other half reported no effect, but all AVG interventions of the studies included were carried out at school. Two further systematic reviews [31,71] supported the findings, although being among the first reviews on the effects of AVGs, quantitative analyses were not conducted due to lack of articles. Lamboglia et al. [71] found that AVG led to an increased PA and cardiorespiratory function and a decreased body fat, with a considerable potential to fight obesity. Leblanc et al. [31] found that AVG helped to attenuate weight gain in overweight and obese participants, including three articles added in the present systematic review. The improvement of cardiometabolic health through AVG was inconclusive due to the small number of articles at the time.

The results seem to establish a positive effect of AVG interventions on BMI when the intervention was longer than 18 weeks and it was supervised and programmed. Home-based AVG interventions may not be appropriated, probably because the duration, frequency and even intensity were lower than that needed to improve BMI or body fat mass.

##### Quantitative Analysis of BMI

As shown in Figure 2, positive effects of the AVG interventions that lasted 18 weeks or more were found for BMI, favoring AVG group compared with control group with no intervention (SMD, −0.590; 95% IC, −1.071, −0.108), showing a moderate SMD effect size. These positive results were not observed for AVG interventions lasting less than 18 weeks (SMD, −0.063; 95% IC, −0.299, 0.174). Heterogeneity among studies for BMI was very low (I^2^ = 0%; *p* < 0.424).

Positive effects have been shown on BMI in favor of the AVG group when the interventions lasted 18 weeks or more, but not when the AVG interventions lasted less. No effects have been shown on percentage of body fat.

#### 3.2.2. Cardiorespiratory Fitness

CRF were evaluated by six articles [35,36,38,48,50,51]. CRF was measured by different tests such as the progressive aerobic cardiovascular endurance run test (PACER) [36,38], the 20-m shuttle run test [35,51], the 3-min step test [48] and the half-mile run test [50]. Three out of six studies reported positive effects on CRF. In the first study reporting effects of AVG in CRF, Lau et al. [51] found improvements in CRF in the AVG group compared with a control group after an intervention with Xbox Kinect (1.58 mL·kg·min, 95% CI: 0.74 to 2.42; *p* = 0.001). Fu et al. [36] performed an 18-week AVG intervention. Authors found an improvement of 20.8 laps in CRF measured by PACER after the AVG intervention (*p* < 0.001), but not in the comparison group. Ye et al. [38] found an increase in CRF in both combined AVG and PE (6.63 laps) and PE only (3.95 laps) groups, with greater improvements for AVG group.

In three out of six studies, positive effects were not found after an AVG intervention. A recent study performed by Gao et al. [48] showed no effects on CRF, measured by 3-min step test, after a 12-week AVG intervention. The participants performed this intervention at home, which can be an explanation for non-significant results. In a study with a sample of 497 participants performed by Azevedo et al. [35], AVG group did not improve their performance in 20-m shuttle run test after a 1-year program with a dance mats AVG sessions in comparison with control group. Probably, more than two hours per week may be needed to obtain benefits in CRF. Ye et al. [50] investigated the change in CRF after an AVG intervention during a school year, where the participants played Xbox Kinect and Nintendo Wii during playtime. Results showed no improvements in CRF after the intervention in both AVG and control groups. These results could be due to a small sample or due to the chosen test for the assessment cardiorespiratory fitness (time to complete a half-mile run) which could be inaccurate.

Four non-controlled trials reported the effects of AVG interventions. Firstly, Owens et al. [63] observed an increase in CRF from 34.3 ± 9.6 to 38.4 ± 8.6 mL·kg·min, measured by the Bruce Ramp Protocol, after 3 months of an AVG intervention with Wii Fit in which participants were encouraged to play at home. Similar results were found by Bethea et al. [64], showing that CRF increased by 2.97 ± 4.99 mL·kg·min (*p* = 0.013), measured by 20-m shuttle run test, after an AVG intervention with DDR. On the other hand, George et al. [72], who used the 6-min walk test to evaluate CRF in 15 children who participated in a 6-week AVG intervention. No significant differences in the distance walked by participants after the AVG intervention was found, indicating that CRF did not change over the study duration. Probably a longer AVG intervention is needed to obtain benefits. Similarly, Gao et al. [73] investigated the effects of a 6-week AVG intervention, showing no effects on CRF after 6 weeks. The results from these four studies should be interpreted with caution as they are non-controlled trials and the sample was always small.

The conclusions of the present review are consistent with those of other reviews. Zeng and Gao [68] included two randomized controlled trials, also included in the present systematic review, which reported positive effects of an AVG intervention in comparison with an exercise group, but these results were unclear due to the lack of studies. Norris et al. [70], reported two studies with improvements in CRF after AVG interventions compared to a control group and one study found no difference between intervention groups. One of these three articles has not been included in this systematic review because the study was conducted in children with autism, being this an exclusion criterion. The interventions of the studies included in the review were carried out at school. In 2014, an overview of systematic reviews was performed by Kari [74] and the evidence did not support the AVG to increase CRF for significant health benefits. Two years later, this overview was updated, and the results were similar [75]. In this updated overview, it was noted that additional high-quality research and systematic reviews concerning AVG are needed, which supports and justifies the present systematic review with meta-analysis.

AVG seems to be a good strategy for improving CRF, but more randomized controlled trials are needed to confirm those benefits. Furthermore, this update of the evidence is necessary as AVGs have been updated, offering more possibilities and more physical demands in exercise sessions using AVG. Thus, in the older articles, AVG interventions used the DDR, the play station with the Eye Toy and the Nintendo Wii, and in the more recent articles, the Nintendo Wii and the Xbox with the Kinect are mainly used. Including AVG strategies into the curricular program such as playtime or PE classes would not be the best alternative. This may be because AVGs reduce the volume of PE rather than being a tool to increase the PA performed in daily life. Instead, AVG included in extracurricular activities could result most beneficial to improve CRF. It is also worth highlighting that no maximal or submaximal incremental cardiopulmonary exercise test with a gas exchange measurement has been performed for evaluating maximal oxygen uptake, despite this being widely recognized as the best single index of aerobic fitness [76,77].

##### Quantitative Analysis of Cardiorespiratory Fitness

**Figure 3:**
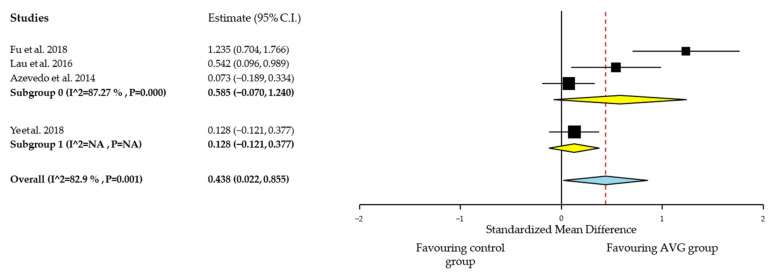
Cardiorespiratory fitness effect sizes for AVG compared with control group. Analysis by control group; Subgroup 1: control group with no intervention; Subgroup 2: exercise control group.

Some articles [48,50] were excluded from meta-analysis due to the use of different measure tools, that reported CRF results in heart rate or time to perform a test, so improvements will be shown by a decrease in the time or heart rate when performing those tests. As shown in Figure 3, positive effects were found for CRF, favoring AVG group compared with control group (SMD, 0.438; 95% IC, 0.022, 0.855), with a moderate SMD effect size. Heterogeneity among studies for CRF was very high (I² = 82.9%; *p* = 0.001). The results also showed a trend towards greater effects when compared to a control group without any intervention (SMD, 0.585; 95% IC, −0.070, 1.240) than when compared to an intervention with exercise (SMD, 0.128; 95% IC, −0.121, 0.377) (Figure 3).

AVG interventions seem to have benefits in CRF but these results should be interpreted with caution because few articles have been included and two articles have not been included. More randomized controlled trials are needed to confirm those positive effects.

#### 3.2.3. Muscular Fitness

From the nineteen controlled trials that focused on the effects of AVG on health-related physical fitness and motor competence in children or adolescents with healthy weight, only two articles evaluated muscular fitness. The first study that investigated the effects of AVG on compiled muscular fitness of children and adolescents with healthy weight was performed by Ye et al. [38] and muscular fitness was evaluated with three tests such as hand-grip dynamometry, push-ups and curl-ups after an AVG intervention using Nintendo Wii and Xbox Kinect during PE class compared with PE only. The between-group difference in the change scores of children’s musculoskeletal fitness was significant, favouring the intervention group. The most recent study was conducted by Çifci et al. [60]. This study is a non-randomized controlled trial that investigated the effect of an 8-week intervention with Xbox Kinect on muscular fitness of adolescents, evaluated by handgrip, leg dynamometry and vertical jump. The children in the AVG group improved muscular fitness, but no differences were found between the AVG group and the control group. The difference between the effects in both studies could be due to two reasons: the length of the intervention (9 months vs. 8 weeks) and the design of the intervention, where the AVG intervention that showed positive effects included more AVG and was performed together with the PE classes in the curricular program of schools. In short, AVG could be useful to develop muscular fitness but positive effects of AVG on muscular fitness are unclear due to the lack of evidence.

Two non-controlled trials evaluated the effects of Nintendo Wii on muscular fitness [63,78]. Owens et al. [63] evaluated the effect of a 3-month AVG intervention using Nintendo Wii at home but results showed no effects on muscular fitness measured by push-ups test. Probably, longer and supervised interventions were needed to obtain benefits on muscular fitness because the time of use of Wii was significantly lower comparing the first six and last six weeks of the intervention, with an average of 12.6 ± 5.5 min per day of Wii use. Smits-Engelsman et al. [78] showed improvements in muscular fitness and in anaerobic fitness with several tests such as long jump, lateral step-up, sit to stand, 10 × 5-m sprint and 10 × 5-m slalom, after a 5-week AVG. In spite of the short duration, there were positive effects probably due to the supervision of professionals, which differentiates it from the previous study.

#### 3.2.4. Motor Competence

Ten studies evaluated the effect of AVG on motor competence and those articles often evaluated also PA. Motor competence was reported by six articles using the TGMD-2 or TGMD-3 [37,46,47,52,53,61], by two articles using the Movement Assessment Battery for children and Bruininks–Oseretsky Test [54] and by three articles using the performance of motor skills such as speed for kicking and throwing, maximum standing long jump distance and hopping [38] or using a balance test with the HUR BT4TM platform [55,56].

Seven out of ten articles reported improvements in motor competence [37,46,47,52,54,55,56]. The most recent article about the effects of AVG in motor competence was performed by Medeiros et al. and the results showed that AVG and PE group improved motor competence, with the control group improving performance on more of the motor skills included in the TGMD-2 than PE group. Fu et al. [37] studied the effect of AVG such as GoNoodles, Adventure to Fitness, and Cosmic Kids Yoga performed in regular school class time on motor competence of preschool children. Children in the AVG group played 30 min of AVG with a frequency of 5 days per week, over 12 weeks while control group had free-play sessions instead of AVG sessions. The results showed that AVG group obtained higher overall TGMD-3 scores and higher step counts than control group (mean difference = 8.7, *p* = 0.019, d = 0.51). Another randomized controlled trial performed by McGann et al. [47] compared the effect of AVG and adapted AVG focused on motor competence improvement. A suite of purpose-built exergames with adaptable features were designed for the intervention group of this study. Four new games were developed using Scratch that was linked to the Kinect^®^ via Kinect2Scratch, each targeting specific locomotor skills (hop, skip, jump and slide). There are three new games: Hop Ball, Jump Ball and Slide Ball; and an existing game: Alien Attack. Participants in both groups played AVG daily for 8 weeks and the results showed improvements in TGMD-2 scores, with a better performance of the group using adapted AVG focused on motor competence. Vernadakis et al. [52] investigated the effects of an 8-week AVG with 60 min per week of Xbox Kinect sessions on object control skills, a part of TGMD, in comparison with a group performing a traditional motor competence training program and in comparison with a no-intervention control group. Great improvements were found for AVG group and traditional motor competence training group but not for control group. Besides, AVG group showed higher enjoyment scores measured by PA enjoyment scale than traditional motor competence training group. Mombarg et al. [54] investigated the benefits of a 6-week intervention performing 5 days per week of 50-min sessions with Nintendo Wii on the motor competence, assessed by the Movement Assessment Battery for children and Bruininks–Oseretsky Test. The results showed that both AVG and the no-intervention control group showed improvements in motor competence. Balance scores of the AVG group improved significantly, whereas those of the control group showed no significant progress. There were significant interaction effects on balance scores, favoring the AVG group. Two articles written by Sheehan et al. [55,56] investigated the effectiveness of AVG on the improvement of balance, assessed by a balance test with the HUR BT4^TM^ platform. One of the interventions of the articles included iDance^TM^, XR Board^TM^ /Lightspace^TM^, and Wii Fit^TM^ Plus [55], while the other article only included Wii Fit^TM^ Plus in the intervention [56]. Both studies compared the AVG group with two control groups: a control group performing ordinal PE curriculum classes and a control group performing PE classes geared toward agility, balance, and coordination improvement. The period of intervention for AVG and control groups lasted 6 weeks and the classes were performed 3 days per week for 34 min each. In both studies, AVG group and the control group performing PE classes geared toward agility, balance, and coordination improvement improved their postural stability significantly compared to the control group performing the curricular PE classes.

Three out of ten articles reported no positive effects or no differences between groups in motor competence [38,53,61]. The first study that investigated the influence of AVG in motor competence in children with healthy weight was performed by Johnson et al. [53]. A 6-week AVG intervention with 5 days per week of 50-min Xbox sessions were performed and found no effects or differences between AVG and control groups. Gao et al. [61], previously described, showed a significant time effect for motor competence (F(1, 52) = 15.61, *p* < 0.01, h²*p* = 0.23) and a significant group by time effect for MVPA (F(1, 52) = 5.06, *p* < 0.02, h²*p* = 0.09). Ye et al. [38] reported results that showed improvements in object control skills in both AVG and PE groups, with PE group demonstrating greater improvements.

The importance of motor competence, together with muscular fitness, lies in their close bidirectional relationship with PA and sports participation, which can improve the physical fitness and body composition of children and adolescents. By increasing muscle strength and motor skills of children and adolescents it is possible that also they will become more physically active, and therefore healthier [11,14].

The use of different tests or batteries for measuring motor competence, the diverse types of control groups and the variety of interventions make it impossible to perform meta-analyses. However, AVG seems to be a promising option in order to help children developing motor skills. A limitation of the results of AVG for improving motor skills was the length of the AVG interventions. Most of the included AVG interventions lasted 8 weeks or less. Only two out of nine articles included an AVG intervention of longer duration, performed by Ye et al. [38] with an intervention´s length of 9 months and Fu et al. [37] with an intervention´s length of 12 weeks.

Two non-controlled trials investigated the effect of an AVG intervention with Nintendo Wii on motor competence assessed by the Movement Assessment Battery for Children [72] or the Bruininks–Oseretsky Test-2 [78]. George et al. [72] found non-significant effects on motor competence, higher in boys than in girls, after 6 weeks of an AVG intervention. Smits-Engelsman et al. [78] showed an improvement in motor competence, specifically in speed and agility (part of the Bruininks–Oseretsky test) but not in balance, after a 5-month AVG intervention with Nintendo Wii.

The results of the present systematic review are consistent with those of other previous reviews with different inclusion and exclusion criteria. The most recent systematic reviews [79,80] were performed including studies with non-typically developing children, developmental coordination disorders and other diseases; one of the reviews even includes adults. Some limitations can be found in these systematic reviews such as the inclusion of non-controlled trials or cross-sectional studies. The results of both systematic reviews were in agreement with the conclusions of the present systematic review and showed that AVG seem to have positive effects on motor skills and could be considered as a potential strategy to develop and improve motor skills. In addition, all 5 studies included in the Norris et al. [70] review reported improvements in motor competence following AVG intervention performed at school, but no studies found differences in motor skill improvements between AVG and other motor skill intervention programs. In the studies included in the review participated children and adolescents without disorders and students with balance disorders.

Overall, the results of AVG on motor competence in children and adolescents with healthy weight are still unclear, but AVG seems to be a promising tool for enhancing motor competence. PA could be a variable to take into account to improve motor competence, considering the direct relation between PA and motor competence [81,82]. AVG increase light-to-moderate PA and energy expenditure [28,29,30,31,32,33], which contributes to improving motor competence. Thus, AVG can be used to enhance motor competence, directly or indirectly through PA.

## 4. Limitations

Some limitations of this review should be recognized. A wide variety of AVG interventions have been included, with different devices and training interventions (duration, frequency, training setting or training dynamic and type of AVG). In addition, the potential risk of bias of some articles was not taken into account when interpreting the results. Lastly, some analyses by subgroups were not performed due to the small number of controlled trials. The present study has also several strengths. To the best of the authors’ knowledge, this is the first meta-analysis to summarize the current research on the effects of AVG on health-related physical fitness and motor competence in healthy-weight children and adolescents. This analysis included not only the effects of AVG on BMI, but on body composition, CRF, muscular fitness and motor competence. This study allowed us to realize that more randomized controlled trials reporting motor competence and muscular fitness results are needed.

## 5. Perspectives

It is necessary to establish the guidelines for an effective intervention using AVGs. It seems that the most effective application of an AVG intervention is sufficient duration (at least 18 weeks) and a structuring and planning of that intervention, in fact, the optimal structure and overall training dose is still unknown being possibly the main factor affecting the effectivity of the AVG. In addition, most of the scientific evidence that has studied AVG interventions combined with traditional exercise lacked a control group to compare the effects, and these AVG interventions seem to be promising, but randomized controlled trials are needed to investigate the effects of these AVG interventions.

Finally, there are few articles studying the effects of an AVG intervention on muscular fitness and motor competence, which are components of the PIT observed by Faigenbaum et al. [14], together with exercise deficit disorder. Randomized controlled trials are recommended in order to investigate the effects of AVG interventions on muscle strength and motor skill to learn about the possibilities of these types of interventions for breaking the vicious circle of PIT.

## 6. Conclusions

AVGs could be a good strategy to control the BMI status and body fat percentage, and to enhance CRF provided that the AVG interventions are supervised and structured, and last at least 18 weeks. Children and adolescents with healthy weight could benefit from AVG to improve motor competence and muscular fitness, but further research is needed to confirm these results because the effects are still unclear.

In conclusion, AVG seem to be an effective tool for improving some components of health-related physical fitness and look like a promising tool also for improvements in motor competence in children and adolescents with healthy weight, which could be important in combating the pediatric inactivity triad. AVGs can even be considered as a complementary alternative to traditional exercise for enhancing the health status during childhood.

## Figures and Tables

**Table 1:** Quality assessment of randomized controlled trials.

Study, Year	R	D	Mi	Me	S	O
Medeiros et al., 2020 [46]	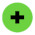	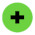	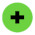	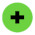	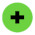	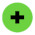
McGann et al., 2019 [47]	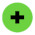	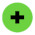	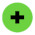	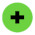	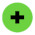	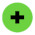
Gao et al., 2019 [48]	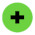	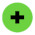	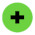	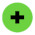	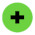	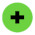
Coknaz et al., 2019 [49]	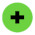	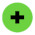	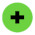	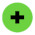	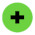	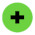
Ye et al., 2019 [50]	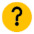	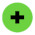	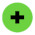	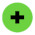	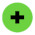	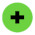
Fu et al., 2018 [37]	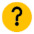	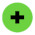	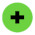	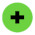	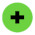	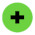
Lau et al., 2017 [51]	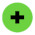	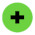	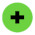	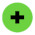	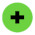	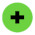
Vernadakis et al., 2015 [52]	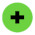	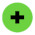	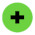	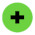	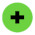	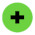
Johnson et al., 2015 [53]	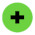	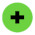	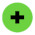	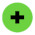	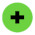	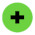
Mombarg et al., 2013 [54]	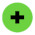	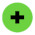	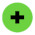	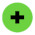	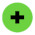	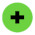
Sheehan et al., 2013 [55]	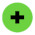	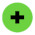	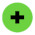	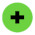	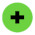	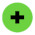
Sheehan et al., 2012 [56]	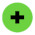	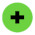	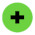	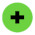	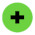	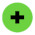
Maloney et al., 2012 [57]	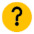	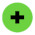	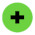	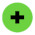	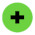	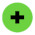
Graves et al., 2010 [58]	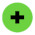	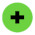	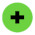	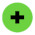	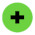	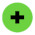
Maloney et al., 2008 [59]	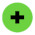	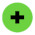	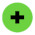	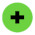	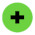	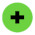

R Bias arising from the randomisation process, D Bias due to deviations from intended interventions, Mi Bias due to missing outcome data, Me Bias in measurement of the outcome, S Bias in selection of the reported result, O Overall risk of bias.

**Table 2:** Quality assessment of non-randomized controlled trials.

Author, Year	Pre-Intervention		At Intervention		Post-Intervention			Overall Risk of Bias
	Bias Due to Confounding	Bias in Selection of Participants into the Study	Bias in Classification of Interventions	Bias Due to Deviations from Intended Interventions	Bias Due to Missing Data	Bias in Measurement of Outcomes	Bias in Selection of the Reported Result	
Çifci et al., 2020 [60]	LOW	LOW	LOW	LOW	LOW	MODERATE	LOW	LOW
Gao et al., 2019 [61]	LOW	LOW	LOW	LOW	LOW	MODERATE	LOW	LOW
Fu et al., 2018 [36]	LOW	LOW	LOW	LOW	LOW	MODERATE	LOW	LOW
Ye et al., 2018 [38]	LOW	LOW	LOW	LOW	LOW	MODERATE	LOW	LOW
Azevedo et al., 2014 [35]	LOW	LOW	LOW	LOW	LOW	MODERATE	LOW	LOW

**Table 3:** Descriptive characteristics of included studies with children with healthy weight.

Study [Ref.]	Participants		Study Design	Intervention	Control	Training	Variables and Test Used	Outcomes
	*n*	Age						
Medeiros et al., 2020 [46]	*n* = 64 male (30) female (34)	9.09 ± 0.75 years	RCT	Xbox Kinect (*n* = 32)	CG: PE curriculum class (*n* = 32)	Period: 9 weeks Frequency: 2 days per week Duration: 45 min per session	MC (TGMD-2)	AVG and CG showed improvements in MC, but AVG showed improvements in more skills of TGMD-2
Çifci et al., 2020 [60]	*n* = 100 male (50) female (50)	12–15 years	Non-RCT	Xbox Kinect (*n* = 50)	No intervention (*n* = 50)	Period: 8 weeks Frequency: 40 min per week	MF (handgrip, leg dynamometry and vertical jump)	AVG improved MF. No differences on MF between AVG and CG were observed.
McGann et al., 2019 [47]	*n* = 40 male (21) female (19)	5–7 years	RCT	AVG focus on MC playing at Scratch with Kinect (*n* = 20)	Traditional AVG (*n* = 20)	Period: 8 weeks Frequency: Daily	MC (TGMD-2)	Improvements on MC for both AVG and CG, and group by time effect with significantly higher scores by AVG.
Gao et al., 2019 [48]	*n* = 32 male (16) female (16)	4.72 ± 0.73 years	RCT	Home-based AVG (LeapTV gaming console) (*n* = 18)	No intervention (*n* = 14)	Period: 12 weeks Frequency: 5 days per week Duration: 30 min per session	BMI CRF (3-Minute Step Test)	No effects for BMI and CRF
Coknaz et al., 2019 [49]	*n* = 106 male (46) female (60)	AVG group: 9.62 ± 1.02 years Control group: 10.31 ± 1.15 years	RCT	Nintendo Wii (*n* = 53)	No intervention	Period: 12 weeks Frequency: 3 days per week Duration: 50–60 min per session	BMI	AVG decreased BMI while CG increased
Gao et al., 2019 [61]	*n* = 56 male (23) female (33)	4.5 ± 0.46 years	Non-RCT	Nintendo Wii and Xbox Kinect (*n* = 20)	No intervention (*n* = 36)	Period: 8 weeks Frequency: 5 days per week Duration: 20 min per session	MC (TGMD-2)	No group effect, but time effect for MC
Ye et al., 2019 [50]	*n* = 81 male (42) female (39)	9.23 ± 0.62 years	RCT	AVG during recess (Nintendo Wii and Xbox Kinect) (*n* = 36)	No intervention (*n* = 45)	Period: School year Frequency: 5 days per week during recess Duration: 50 min per session	CRF (Half-mile run test)	No effects for CRF
Fu et al., 2018 [36]	*n* = 65 male and female	11.6 ± 0.5 years	Non-RCT	AVG (GoNoodle, Adventure to Fitness, and Cosmic Kids Yoga) in regular school class time (*n* = 33)	Five 30-min of free-play sessions (*n* = 32)	Period: 18 weeks Frequency: 3 days per week Duration: 30 min per session	CRF (20-m PACER)	AVG showed a higher CRF than CG
Fu et al., 2018 [37]	*n* = 65 male (34) female (31)	4.9 ± 0.7 years	RCT	AVG (GoNoodles, Adventure to Fitness, and Cosmic Kids Yoga) in regular school class time (*n* = 36)	Five 30-min of free-play sessions (*n* = 29)	Period: 12 weeks Frequency: 5 days per week Duration: 30 min per session	MC (TGMD-3)	AVG showed higher increase in TGMD-3 score than CG
Ye et al., 2018 [38]	*n* = 250 male and female	8.27 ± 0.70 years	Non-RCT	125 min of AVG (Nintendo Wii and Xbox Kinect) + PE (*n* = 135)	125 min of PE only (5 classes per week) (*n* = 115)	Period: 9 month Frequency: 3 PE classes and 2 exergaming sessions per week Duration: 125 min	BMI HRF (FITNESSGRAM protocols for PACER) MF (hand-grip dynamometry, push-ups and curl-ups) MC (speed for kicking and throwing, maximum standing long jump distance and hopping)	AVG improved BMI, CRF, MF and OCS, while CG improved CRF and object control skills but worsened MF and BMI.
Lau et al., 2016 [51]	*n* = 80 male (25) female (55)	9.23 ± 0.52 years	RCT	Xbox Kinect (*n* = 40)	No intervention (*n* = 40)	Period: 12 weeks Frequency: twice a week after school Duration: 60 min per day	BMI CRF (20-m shuttle run test)	BMI increased in AVG and CRF increased in AVG and CG, with higher improvements in AVG
Vernadakis et al., 2015 [52]	*n* = 66 males and females	6.35 ± 0.73 years	RCT	Xbox Kinect (*n* = 22)	EXE: traditional motor competence training program (*n* = 22) and no intervention (*n* = 22)	Period: 8 weeks Frequency and duration: Non-reported	MSC (OCS with TGMD-2)	AVG and EXE showed improvements in OCS. CG2 showed no improvements
Johnson et al., 2015 [59]	*n* = 36 male (53%) female (47%)	6–10 years	RCT	Xbox Kinect (*n* = 19; 7.9 ± 1.5 years)	No intervention (*n* = 17; 8.0 ± 1.2 years)	Period: 6 weeks Frequency: 5 days per week Duration: 50 min in lunchtime	MSC (OCS with TGMD-3)	No effects on OCS or differences between groups for AVG
Azevedo et al., 2014 [35]	*n* = 497 male and female	11–13 years old	Non-RCT	Dance Mat Exergaming (*n* = 280; 11.2 ± 0.4 year; 63.9% female)	No intervention (*n* = 217, 11.3 ± 0.4 years; 64.5% female)	Period: 12 months Frequency: 2 h per week	BMI %BF (DXA) CRF (20-m shuttle run test)	AVG showed a positive effect on weight, BMI and %BF compared to CG, but not on CRF
Mombarg et al., 2013 [54]	*n* = 29 male (23) female (6)	7–12 years	RCT	Nintendo Wii (Wii-balance board with the Wii-fit-plus) (*n* = 15)	No intervention (*n* = 14)	Period: 6 weeks Frequency: 3 days per week Duration: 30 min per session	MC (movement assessment battery for children and Bruininks–Oseretsky Test)	Both AVG and CG showed improvements in MC. Balance scores of the AVG improved significantly, whereas those of the control group showed no significant progress. There were significant interaction effects on balance scores, favoring the AVG.
Sheehan et al., 2013 [55]	*n* = 61 male (33) female (28)	9–10 years	RCT	iDance^TM^, XR Board^TM^ /Lightspace^TM^, and Wii Fit^TM^ Plus. (*n* = 21)	CG1: PE curriculum class (*n* = 21) CG2: PE geared toward agility, balance, and coordination (ABC) improvement (*n* = 19)	Period: 6 weeks Frequency: 4–5 days per week Duration: 34 min per session	MC (balance test with the HUR BT4^TM^ platform)	AVG and the ABC PE (CG2) improved their postural stability significantly compared to those in the curricular PE class.
Sheehan et al., 2012 [56]	*n* = 65 male (29) female (36)	9–10 years	RCT	Wii Fit+™ (*n* = 22)	CG1: PE curriculum class (*n* = 21) CG2: PE geared toward agility, balance, and coordination (ABC) improvement (*n* = 22)	Period: 6 weeks Frequency: 3 days per week Duration: 34 min per session	MC (balance test with the HUR BT4^TM^ platform)	AVG and the ABC PE (CG2) improved the balance compared to those in the curricular PE class. Improvement were not significantly different from CG2.
Maloney et al., 2012 [57]	*n* = 58 male (71%) female (29%)	13.7 ± 0.6 years	RCT	Playstation 2 (In the Groove) (*n* = 29)	No intervention (*n* = 29)	Period: 10 and 20 weeks Frequency: 4–5 school days per week Duration: 10 min per session	BMI	AVG decreased BMI more than CG
Graves et al., 2010 [58]	*n* = 58 male (39) female (19)	9.2 ± 0.5 years	RCT	Nintendo Wii jOG (*n* = 29)	No intervention (*n* = 29)	Period: 6 and 12 weeks Frequency and duration: Non-reported home-based	BMI FM (DXA)	No effects for BF or BMI
Maloney et al., 2008 [59]	*n* = 60 male (30) female (30)	7.5 ± 0.5 years	RCT	DDR (*n* = 40)	No intervention (*n* = 20)	Period: 28 weeks Home-based	BMI	No changes in BMI

%BF body fat percentage, AVG active video games, BMI body mass index, CG control group, CRF cardiorespiratory fitness, DXA dual-energy X-ray absorptiometry, EXE exercise, FFM fat-free mass, FM fat mass, FPG free-play group, HRF health-related fitness, MF musculoskeletal fitness, min minutes, MSC motor skills competence, OCS object control skills, PACER progressive aerobic cardiovascular endurance run, PE physical education, RCT randomized controlled trial, TGMD test of gross motor development.

**Table 4:** Effect sizes and heterogeneity of findings for studies comparing AVG intervention versus control group.

Variable	*N*	Hedges’g Effect Size	95 % CI	*p* Value	I²
BMI	7	−0.291	−0.631; 0.049	0.000	78.47%
CRF	4	0.438	0.022; 0.855	0.001	82.9%

BMI body mass index, CRF cardiorespiratory fitness.

## Supplementary Materials

The following are available online at https://www.mdpi.com/article/10.3390/ijerph18136965/s1, Table S1: PRISMA 2020 item Checklist, Table S2: Descriptive characteristics of included non-controlled trials.

## References

[1] Ortega F.B., Ruiz J.R., Castillo M.J., Sjöström M. (2008). Physical fitness in childhood and adolescence: A powerful marker of health. Int. J. Obes..

[2] Chen W., Hammond-Bennett A., Hypnar A., Mason S. (2018). Health-related physical fitness and physical activity in elementary school students. BMC Public Health.

[3] Pahkala K., Hernelahti M., Heinonen O.J., Raittinen P., Hakanen M., Lagström H., Viikari J.S.A., Rönnemaa T., Raitakari O.T., Simell O. (2013). Body mass index, fitness and physical activity from childhood through adolescence. Br. J. Sports Med..

[4] Blair S.N., Cheng Y., Holder J.S. (2001). Is physical activity or physical fitness more important in defining health benefits?. Sci. Sport. Exerc..

[5] Tomkinson G.R., Carver K.D., Atkinson F., Daniell N.D., Lewis L.K., Fitzgerald J.S., Lang J., Ortega F.B. (2017). European normative values for physical fitness in children and adolescents aged 9–17 years: Results from 2 779 165 Eurofit performances representing 30 countries. Br. J. Sports Med..

[6] Micheli L., Mountjoy M., Engebretsen L., Hardman K., Kahlmeier S., Lambert E., Ljungqvist A., Matsudo V., McKay H., Sundberg C.J. (2011). Fitness and health of children through sport: The context for action. Br. J. Sports Med..

[7] Ahluwalia N., Dalmasso P., Rasmussen M., Lipsky L., Currie C., Haug E., Kelly C., Damsgaard M.T., Due P., Tabak I. (2015). Trends in overweight prevalence among 11-, 13- and 15-year-olds in 25 countries in Europe, Canada and USA from 2002 to 2010. Eur. J. Public Health.

[8] Nittari G., Scuri S., Petrelli F., Pirillo I., Di Luca N.M., Grappasonni I. (2019). Fighting obesity in children from European World Health Organization member states. Epidemiological data, medical-social aspects, and prevention programs. La Clin. Ter..

[9] Westerterp K.R. (2018). Changes in physical activity over the lifespan: Impact on body composition and sarcopenic obesity. Obes. Rev..

[10] García-Hermoso A., Alonso-Martinez A.M., Ramírez-Vélez R., Izquierdo M. (2019). Effects of Exercise Intervention on Health-Related Physical Fitness and Blood Pressure in Preschool Children: A Systematic Review and Meta-Analysis of Randomized Controlled Trials. Sports Med..

[11] Poitras V.J., Gray C., Borghese M.M., Carson V., Chaput J.-P., Janssen I., Katzmarzyk P., Pate R.R., Gorber S.C., Kho M. (2016). Systematic review of the relationships between objectively measured physical activity and health indicators in school-aged children and youth. Appl. Physiol. Nutr. Metab..

[12] Bull F.C., Al-Ansari S.S., Biddle S., Borodulin K., Buman M.P., Cardon G., Carty C., Chaput J.-P., Chastin S., Chou R. (2020). World Health Organization 2020 guidelines on physical activity and sedentary behaviour. Br. J. Sports Med..

[13] Guthold R., Stevens G.A., Riley L.M., Bull F.C. (2020). Global trends in insufficient physical activity among adolescents: A pooled analysis of 298 population-based surveys with 1·6 million participants. Lancet Child Adolesc. Health.

[14] Faigenbaum A.D., Rial Rebullido T., MacDonald J.P. (2018). Pediatric Inactivity Triad: A Risky PIT. Curr. Sports Med. Rep..

[15] Moliner-Urdiales D., Ruiz J.R., Ortega F.B., Jiménez-Pavón D., Vicente-Rodriguez G., Rey-López J.P., Martínez-Gómez D., Casajús J.A., Mesana M.I., Marcos A. (2010). Secular trends in health-related physical fitness in Spanish adolescents: The AVENA and HELENA Studies. J. Sci. Med. Sport.

[16] Cohen D., Voss C., Taylor M., Delextrat A., Ogunleye A., Sandercock G. (2011). Ten-year secular changes in muscular fitness in English children. Acta Paediatr. Int. J. Paediatr..

[17] Albon H.M., Hamlin M.J., Ross J.J. (2010). Secular trends and distributional changes in health and fitness performance variables of 10-14-year-old children in New Zealand between 1991 and 2003. Br. J. Sports Med..

[18] Faigenbaum A.D., Bruno L.E. (2017). A fundamental approach for treating pediatric dynapenia in kids. ACSM Health Fit. J..

[19] Smith J.J., Eather N., Weaver R.G., Riley N., Beets M.W., Lubans D.R. (2019). Behavioral Correlates of Muscular Fitness in Children and Adolescents: A Systematic Review. Sports Med..

[20] Fühner T., Kliegl R., Arntz F., Kriemler S., Granacher U. (2021). An Update on Secular Trends in Physical Fitness of Children and Adolescents from 1972 to 2015: A Systematic Review. Sports Med..

[21] Lai S.K., Costigan S.A., Morgan P.J., Lubans D.R., Stodden D.F., Salmon J., Barnett L.M. (2014). Do School-Based Interventions Focusing on Physical Activity, Fitness, or Fundamental Movement Skill Competency Produce a Sustained Impact in These Outcomes in Children and Adolescents? A Systematic Review of Follow-Up Studies. Sports Med..

[22] Jaakkola T., Yli-Piipari S., Huotari P., Watt A., Liukkonen J. (2016). Fundamental movement skills and physical fitness as predictors of physical activity: A 6-year follow-up study. Scand. J. Med. Sci. Sports.

[23] Cattuzzo M.T., Henrique R.D.S., Ré A.H.N., de Oliveira I.S., Melo B.M., Moura M.D.S., de Araújo R.C., Stodden D. (2016). Motor competence and health related physical fitness in youth: A systematic review. J. Sci. Med. Sport.

[24] Milne N., Leong G.M., Hing W. (2016). The relationship between children’s motor proficiency and health-related fitness. J. Paediatr. Child Health.

[25] Lubans D.R., Morgan P.J., Cliff D.P., Barnett L.M., Okely A.D. (2010). Fundamental Movement Skills in Children and Adolescents Review of Associated Health Benefits. Sport. Med..

[26] American College of Sport Medicine Exercise is Medicine: The Power of Physical Activity. https://www.exerciseismedicine.org/support_page.php/physical-activity-health-impact/.

[27] Beedie C., Mann S., Jimenez A., Kennedy L., Lane A.M., Domone S., Wilson S., Whyte G. (2016). Death by effectiveness: Exercise as medicine caught in the efficacy trap!. Br. J. Sports Med..

[28] Peng W., Lin J.-H., Crouse J. (2011). Is Playing Exergames Really Exercising? A Meta-Analysis of Energy Expenditure in Active Video Games. Cyberpsychol. Behav. Soc. Netw..

[29] Sween J., Wallington S.F., Sheppard V., Taylor T., Llanos A., Adams-Campbell L.L. (2014). The Role of Exergaming in Improving Physical Activity: A Review. J. Phys. Act. Health.

[30] Gao Z., Chen S., Pasco D., Pope Z. (2015). A meta-analysis of active video games on health outcomes among children and adolescents. Obes. Rev..

[31] Leblanc A.G., Chaput J.-P., McFarlane A., Colley R.C., Thivel D., Biddle S.J.H., Maddison R., Leatherdale S.T., Tremblay M.S. (2013). Active Video Games and Health Indicators in Children and Youth: A Systematic Review. PLoS ONE.

[32] Biddiss E., Irwin J. (2010). Active Video Games to Promote Physical Activity in Children and Youth A Systematic Review. Arch. Pediatr. Adolesc. Med..

[33] Barnett A., Cerin E., Baranowski T. (2011). Active Video Games for Youth: A Systematic Review. J. Phys. Act. Health.

[34] Comeras-Chueca C., Villalba-Heredia L., Pérez-Llera M., Lozano-Berges G., Marín-Puyalto J., Vicente-Rodríguez G., Matute-Llorente Á., Casajús J.A., González-Agüero A. (2020). Assessment of Active Video Games’ Energy Expenditure in Children with Overweight and Obesity and Differences by Gender. Int. J. Environ. Res. Public Health.

[35] Azevedo L.B., Watson D.B., Haighton C., Adams J. (2014). The effect of dance mat exergaming systems on physical activity and health—related outcomes in secondary schools: Results from a natural experiment. BMC Public Health.

[36] Fu Y., Burns R.D. (2018). Effect of an Active Video Gaming Classroom Curriculum on Health-Related Fitness, School Day Step Counts, and Motivation in Sixth Graders. J. Phys. Act. Health.

[37] Fu Y., Burns R.D., Constantino N., Zhang P. (2018). Differences in Step Counts, Motor Competence, and Enjoyment Between an Exergaming Group and a Non-Exergaming Group. Games Health J..

[38] Ye S., Lee J.E., Stodden D.F., Gao Z. (2018). Impact of Exergaming on Children’s Motor Skill Competence and Health-Related Fitness: A Quasi-Experimental Study. J. Clin. Med..

[39] De Onis M., Onyango A.W., Borghi E., Siyam A., Nishida C., Siekmann J. (2007). Development of a WHO growth reference for school-aged children and adolescents. Bull. World Health Organ..

[40] Higgins J.P.T., Green S. (2011). Cochrane Handbook for Systematic Reviews of Interventions Version 5.1.0 [Updated March 2011].

[41] Page M.J., McKenzie J.E., Bossuyt P.M., Boutron I., Hoffmann T.C., Mulrow C.D., Shamseer L., Tetzlaff J.M., Akl E.A., Brennan S.E. (2021). The PRISMA 2020 statement: An updated guideline for reporting systematic reviews. BMJ.

[42] Moher D., Shamseer L., Clarke M., Ghersi D., Liberati A., Petticrew M., Shekelle P., Stewart L.A., Estarli M., Barrera E.S.A. (2016). Preferred reporting items for systematic review and meta-analysis protocols (PRISMA-P) 2015 statement. Rev. Esp. Nutr. Hum. Diet..

[43] Sterne J.A.C., Savović J., Page M.J., Elbers R.G., Blencowe N.S., Boutron I., Cates C.J., Cheng H.-Y., Corbett M.S., Eldridge S.M. (2019). RoB 2: A revised tool for assessing risk of bias in randomised trials. BMJ.

[44] Sterne J.A., Hernán M.A., Reeves B.C., Savović J., Berkman N.D., Viswanathan M., Henry D., Altman D.G., Ansari M.T., Boutron I. (2016). ROBINS-I: A tool for assessing risk of bias in non-randomised studies of interventions. BMJ.

[45] Higgins J.P.T., Altman D.G., Gøtzsche P.C., Jüni P., Moher D., Oxman A.D., Savović J., Schulz K.F., Weeks L., Sterne J.A.C. (2011). The Cochrane Collaboration’s tool for assessing risk of bias in randomised trials. BMJ.

[46] Medeiros P., Felden É.P.G., Zequinão M.A., Cordeiro P.C., de Freitas K.T.D., dos Santos J.O.L., Cardoso F.L. (2020). Positive effect of a motor intervation program with exergames: A blind randomized trial. Int. J. Game Based Learn..

[47] McGann J., Issartel J., Hederman L., Conlan O. (2019). Hop.Skip.Jump.Games: The effect of “principled” exergameplay on children’s locomotor skill acquisition. Br. J. Educ. Technol..

[48] Gao Z., Lee J.E., Zeng N., Pope Z.C., Zhang Y., Li X. (2019). Home-Based Exergaming on Preschoolers’ Energy Expenditure, Cardiovascular Fitness, Body Mass Index and Cognitive Flexibility: A Randomized Controlled Trial. J. Clin. Med..

[49] Coknaz D., Mirzeoglu A.D., Atasoy H.I., Alkoy S., Coknaz H., Goral K. (2019). A digital movement in the world of inactive children: Favourable outcomes of playing active video games in a pilot randomized trial. Eur. J. Nucl. Med. Mol. Imaging.

[50] Ye S., Pope Z.C., Lee J.E., Gao Z. (2019). Effects of school-based exergaming on urban children’s physical activity and cardiorespiratory fitness: A quasi-experimental study. Int. J. Environ. Res. Public Health.

[51] Lau P.W.C., Wang J.J., Maddison R. (2016). A Randomized-Controlled Trial of School-Based Active Videogame Intervention on Chinese Children’s Aerobic Fitness, Physical Activity Level, and Psychological Correlates. Games Health J..

[52] Vernadakis N., Papastergiou M., Zetou E., Antoniou P. (2015). The impact of an exergame-based intervention on children’s fundamental motor skills. Comput. Educ..

[53] Johnson T.M., Ridgers N.D., Hulteen R.M., Mellecker R.R., Barnett L.M. (2016). Does playing a sports active video game improve young children’s ball skill competence?. J. Sci. Med. Sport.

[54] Mombarg R., Jelsma D., Hartman E. (2013). Effect of Wii-intervention on balance of children with poor motor performance. Res. Dev. Disabil..

[55] Sheehan D.P., Katz L. (2013). The effects of a daily, 6-week exergaming curriculum on balance in fourth grade children. J. Sport Health Sci..

[56] Sheehan D.P., Katz L. (2012). The impact of a six week exergaming curriculum on balance with grade three school children using the wii FIT+TM. Int. J. Comput. Sci. Sport.

[57] Maloney A.E., Stempel A., Wood M.E., Patraitis C., Beaudoin C. (2012). Can Dance Exergames Boost Physical Activity as a School-Based Intervention?. Games Health J..

[58] Graves L.E.F., Ridgers N.D., Atkinson G., Stratton G. (2010). The Effect of Active Video Gaming on Children’s Physical Activity, Behavior Preferences and Body Composition. Pediatr. Exerc. Sci..

[59] Maloney A.E., Bethea T.C., Kelsey K.S., Marks J.T., Paez S., Rosenberg A.M., Catellier D.J., Hamer R.M., Sikich L. (2008). A Pilot of a Video Game (DDR) to Promote Physical Activity and Decrease Sedentary Screen Time. Obesity.

[60] Cifci C., Baspinar S.G. (2020). The Effects of Active Video Games on Strength, Vertical Jumping and Flexibility in Children Aged 12 to 15 Years Old. Int. J. Appl. Exerc. Physiol..

[61] Gao Z., Zeng N., Pope Z.C., Wang R., Yu F. (2019). Effects of exergaming on motor skill competence, perceived competence, and physical activity in preschool children. J. Sport Health Sci..

[62] De Brito-Gomes J.L., Perrier-Melo R.J., De Oliveira S.F.M., Guimarães F.J.D.S.P., Costa M.D.C. (2016). Physical Effort, Energy Expenditure, and Motivation in Structured and Unstructured Active Video Games: A Randomized Controlled Trial. Hum. Mov..

[63] Owens S.G., Garner J.C., Loftin J.M., van Blerk N., Ermin K. (2011). Changes in Physical Activity and Fitness after 3 Months of Home Wii Fit Use. J. Strength Cond. Res..

[64] Bethea T.C., Berry D., Maloney A.E., Sikich L. (2012). Pilot Study of an Active Screen Time Game Correlates with Improved Physical Fitness in Minority Elementary School Youth. Games Health J..

[65] Gao Z., Zeng N., McDonough D.J., Su X. (2020). A Systematic Review of Active Video Games on Youth’s Body Composition and Physical Activity. Int. J. Sports Med..

[66] Hernández-Jiménez C., Sarabia R., Paz-Zulueta M., Paras-Bravo P., Pellico A., Azcona L.R., Blanco C., Madrazo M., Agudo M.J., Sarabia C. (2019). Impact of Active Video Games on Body Mass Index in Children and Adolescents: Systematic Review and Meta-Analysis Evaluating the Quality of Primary Studies. Int. J. Environ. Res. Public Health.

[67] Oliveira C.B., Pinto R.Z., Saraiva B.T.C., Tebar W.R., Delfino L.D., Franco M.R., Silva C.C.M., Christofaro D.G.D. (2020). Effects of active video games on children and adolescents: A systematic review with meta-analysis. Scand. J. Med. Sci. Sports.

[68] Zeng N., Gao Z. (2016). Exergaming and obesity in youth: Current perspectives. Int. J. Gen. Med..

[69] Gao Z., Chen S. (2014). Are field-based exergames useful in preventing childhood obesity? A systematic review. Int. Assoc. Study Obes..

[70] Norris E., Hamer M., Stamatakis E. (2016). Active Video Games in Schools and Effects on Physical Activity and Health: A Systematic Review. J. Pediatr..

[71] Lamboglia C.M.G.F., Da Silva V.T.B.L., Filho J.E.D.V., Pinheiro M.H.N.P., Munguba M., Júnior F.V.I.S., De Paula F.A.R., Da Silva C.A.B. (2013). Exergaming as a Strategic Tool in the Fight against Childhood Obesity: A Systematic Review. J. Obes..

[72] George A.M., Rohr L.E., Byrne J. (2016). Impact of Nintendo Wii Games on Physical Literacy in Children: Motor Skills, Physical Fitness, Activity Behaviors, and Knowledge. Sports.

[73] Gao Z., Lee J.E., Pope Z., Zhang D. (2016). Effect of Active Videogames on Underserved Children’s Classroom Behaviors, Effort, and Fitness. Games Health J..

[74] Kari T. (2014). Can Exergaming Promote Physical Fitness and Physical Activity?. Int. J. Gaming Comput. Simul..

[75] Kari T. (2016). Promoting Physical Activity and Fitness with Exergames. Transforming Gaming and Computer Simulation Technologies across Industries.

[76] Van Brussel M., Bongers B.C., Hulzebos E.H., Burghard M., Takken T. (2019). A Systematic Approach to Interpreting the Cardiopulmonary Exercise Test in Pediatrics. Pediatr. Exerc. Sci..

[77] Armstrong N., Welsman J., Winsley R. (1996). Is Peak VO2a Maximal Index of Children’s Aerobic Fitness?. Int. J. Sport. Med..

[78] Smits-Engelsman B.C., Jelsma L.D., Ferguson G.D. (2017). The effect of exergames on functional strength, anaerobic fitness, balance and agility in children with and without motor coordination difficulties living in low-income communities. Hum. Mov. Sci..

[79] De Medeirosa P., Capistranoa R., Almeida M., Aparecida S., Silva T., Cardoso F. (2017). Exergames as a tool for the acquisition and development of motor skills and abilities: A systematic review. Rev. Paul. Pediatr..

[80] Page Z.E., Barrington S., Edwards J., Barnett L.M. (2017). Do active video games benefit the motor skill development of non-typically developing children and adolescents: A systematic review. J. Sci. Med. Sport.

[81] De Meester A., Maes J., Stodden D., Cardon G., Goodway J., Lenoir M., Haerens L. (2016). Identifying profiles of actual and perceived motor competence among adolescents: Associations with motivation, physical activity, and sports participation. J. Sports Sci..

[82] Bardid F., De Meester A., Tallir I., Cardon G., Lenoir M., Haerens L. (2016). Configurations of actual and perceived motor competence among children: Associations with motivation for sports and global self-worth. Hum. Mov. Sci..

